# A Finite Element Model of Electrode Placement During Stimulus Evoked Electromyographic Monitoring of Iliosacral Screw Insertion

**DOI:** 10.2174/1874325000802010033

**Published:** 2008-03-10

**Authors:** M.A Kopec, B.R Moed, D.W Barnett

**Affiliations:** 1 Department of Biomedical Engineering, Saint Louis University, St. Louis, MO, USA; 2 Department of Orthopaedic Surgery, Saint Louis University School of Medicine, St. Louis, MO, USA

**Keywords:** Finite element modeling, spinal nerve roots, stimulus-evoked electromyography.

## Abstract

Pelvic ring fractures that occur as a result of substantial orthopedic trauma are frequently repaired using iliosacral screws to stabilize the fracture. Stimulus evoked electromyography, using pulsed current stimuli provided through the drill bit cathode, has been advocated to prevent nerve root injury during iliosacral screw insertion. Our objective was to examine the effects of anode location, drill bit position, and anatomical structure on the nerve monitoring technique. A three-dimensional finite element model was constructed from computed tomography data to evaluate the effectiveness of five anode locations at four stations of drill bit insertion. Results indicate that the anode location should be at the midline or on the side contralateral to drill bit insertion. Locating the anode at other positions, such that the nerve root is outside of the primary electromagnetic field, leads to an attenuated electromyographic response that will ultimately lead to the failure of the monitoring technique.

## INTRODUCTION

Pelvic ring fractures resulting from motor vehicle accidents or substantial falls are a relatively common form of orthopaedic trauma. When the posterior portion of the ring is displaced and/or unstable, a common method of restoring mechanical stability is the insertion of iliosacral screws. However, the proximity of the ideal screw trajectory to the fifth lumbar (L5) and first sacral nerve roots (S1), as well as the spinal canal, make neural tissue injury a potential hazard. Screw trajectory deviation of as little as 4° has been reported to direct the screw into the S1 nerve root [1]. To assist in proper screw placement, guide-wires are inserted under fluoroscopic guidance followed by the screws. However, fluoroscopy alone has not been shown to be sufficient for avoiding nerve root injury, which has been reported to range from 1% to as many as 18% of cases [2, 3]. Newer image navigation systems, such as fluoroscopy-based 3-D imaging and fluoroscopically registered computer-assisted surgery techniques, have yet to demonstrate any improvement over standard fluoroscopic techniques [4, 5]. As an alternative to these imaging methods, a simple technique to reduce the risk of surgical complications based on an evoked electromyographic (EMG) response may be used to determine the proximity of the drill bit to the neural structure [3, 6-8].

The drill bit’s depolarizing stimulus, created by a small current (< 50 mA searching current delivered in 0.2 ms duration pulses three times per second) between the tip of the dill-bit and a reference electrode [7, 8], causes action potential propagation from the nervous tissue towards the muscles they innervate. The magnitude of the stimulus required to induce a 20 mV EMG response (the current threshold) can be used to infer the distance between the tip of the drill bit and the neural structure intraoperatively. The location of the reference electrode relative to the drill bit determines the path which current flows through the body, and therefore the potential at any point on the nervous structure. The significance of anode location on the effectiveness of the nerve monitoring technique has been identified as an important variable [6, 9, 10]. This study aims to computationally investigate the effect of anode location on the current density observed in the first sacral nerve root (S1) using finite element modeling (FEM). This method has been employed extensively when modeling electrical activity in the torso and cranium, but not in the midsection [11-14]. Examination of different reference electrode locations is straightforward using a FEM. The modeling approach provides a lucid illustration of the current emanating from the cathode that is easily reconfigurable for examining multiple configurations. The insight provided by the FEM provides a clear scientific justification for the selection of an optimal anode location.

## MATERIALS AND METHODOLOGY

Ninety-three axial computed tomography (CT) slices from the level of the fourth lumbar vertebrae to the diaphysis of the femur were obtained from a healthy female subject with 0.08 cm x 0.08 cm x 0.25 cm resolution. The voxel data were segmented by tissue type in a commercially available software package (Amira 3.1, Mercury Computer Systems Inc., Chelmsford, MA). These data were used to create tissue surfaces, which were compiled to construct a solid tetrahedral mesh in the same software environment. The number of elements of each tissue type and their conductivity values, drawn from well-accepted studies, are shown in Table **1**. The final mesh was imported into the FEM solver for analysis (Algor V16, Algor Inc., Pittsburgh, PA). The changes in tissue impedance that occur with changes in frequency were ignored for this analysis since the bandwidth of the stimulus is approximately 5 kHz and reported tissue impedances show little change over the low frequency ranges considered here [13]. Anisotropy of the tissue conductance was also ignored. These assumptions allowed the electrostatics module in Algor, which solves the governing equation (Poisson) over the entire computational domain, to be used to calculate the current density throughout the model. The anode was assigned the ground potential and the stimulus current was generated in elements located at the end of the 6.5 mm diameter drill bit (representative of commercially available systems). Analysis time was approximately ten minutes on a 3.4 GHz Dell Dimension 370 (Dell Inc., Round Rock, TX). The number of elements in each tissue and their conductivity values are shown in Table **1**.

The current density in the S1 nerve root was studied in two basic configurations. First, the five anode locations depicted in Fig. (**1**) were examined with the drill bit (cathode) in the normal final resting position (station III). The five anode locations were the contralateral anterior superior iliac spine (CASIS), the ipsilateral anterior superior iliac spine (IASIS), the dorsal and ventral midlines (DM, VM) at the level of the umbilicus, and 2 cm anterior to the insertion site (IS). Regression data from animal studies [8] were used to determine the applied current (7.28 mA) based on a 5 mm perpendicular distance from the drill bit to the nerve root, in the transverse plane, with the anode at the VM location.

Second, using the ventral midline anode location, models were created to examine four stations of screw insertion: station I: the sacroiliac joint; station II: prior to the tip of the drill bit crossing anteromedial to the nerve root; station III: the normal final screw resting position (drill bit tip located approximately on the midline of the body); and station IV: the drill bit tip approaching the contralateral nerve root. As with the anode location comparison, the minimum perpendicular drill bit to nerve root distance was 5 mm and all models were subjected to the same stimulus level of 7.28 mA. Finally, with the drill bit at station III, the insertion pathway was adjusted such that the drill bit passed at a distance of 2 mm, 5 mm or 10 mm from the nerve root as measured orthogonal to the direction of the screw insertion, see Fig. (**1**). Once again, the anode was located at the VM location and the stimulus level was either held constant at 8 mA, or selected, based on the distance [8], as 4.71 mA for the 2mm model, 7.28 mA for the 5 mm model, and 10.78 mA for the 10 mm model.

The nerve root current density magnitude was exported for analysis in Matlab (The Mathworks, Natick, MA). The maximum current density magnitude (|Jmax|) at an axial cross section of the ipsilateral S1 nerve root was examined under each of the test conditions described previously. It is important to note that an increase in peak |Jmax| coincides with a lower current threshold for evoking an EMG response. Although not presented here, the sensitivity of the model was assessed in order to eliminate any model-dependent results due to mesh size and conductivity parameters. Specifically, the number of tetrahedral elements for each model was selected such that further increasing the mesh density did not impact the resultant current density (<1%); and, altering any single tissue conductivity value by ±20% produced only minor changes in the peak current density (<10%) and no qualitative change in the distribution of current.

## RESULTS

The current density magnitude distribution in the L5, S1, and S2 nerve roots is shown in Fig. (**2**) for the drill bit located at station III at a perpendicular distance of 5 mm from the nerve, and the VM anode location. Under these test conditions, the peak current density occurs posterior and superior to the drill bit. While the magnitude of the response varies with anode location, the current density distribution throughout the ipsilateral S1 nerve root displays a similar pattern as shown in Fig. (**3a**) for three anode locations (IS, VM, DM). The maximum peak current density was produced in the region superior to the plane of insertion (~1 cm) with the anode at location DM. In addition to the peak |Jmax|, the DM anode location also produced an increased current density in the portion of the nerve root 1 cm inferior to the transverse plane of drill bit insertion. Overall, as the anode moved counter clockwise away from the insertion site to IASIS to VM to CASIS to DM, the peak |Jmax| increased. Relative to the DM peak |Jmax|, the other locations were attenuated by 23%, 26%, 67% and 87% respectively for the CASIS, VM, IASIS, and IS positions (see Fig (**3b**)). In addition to the peak values, Fig. (**3c**) depicts a backward-cumulative histogram that illustrates the fraction of S1 versus minimum current density. For example, with the anode in the IS location, 29% of the nerve root displayed a current density of at least 0.01 mA/cm^2^ while 87.5% of the S1 tissue displayed the same current density for the IASIS anode location. As the curves shift to the right, there is an increase in the fraction of S1 nerve root displaying a particular current density as well as an increase in peak current density level, thus leading to an overall increase in the likelihood of evoking an EMG response.

Simulation results of the drill bit motion along the insertion path (with the anode at the VM location) are shown in Fig. (**4a**,**b**) for the ipsilateral S1 nerve root. Moving from station I to II increased the peak |Jmax| by 77%. Conversely, moving from II to III and III to IV decreased the peak |Jmax| by 17 % and 3% respectively. Drill bit placement at station I also increased the |Jmax| in the distal portion of the nerve. Fig. (**4c**) reveals an increase in the portion of contralateral S1 nerve root that meets, or exceeds, a given threshold value as the anode location is changed from VM to CASIS.

The effect of varying the perpendicular distance of the final screw resting position, i.e., translation of the screw insertion path towards the anterior cortex of the sacrum as shown in Fig. (**1**), is summarized in Table **2**. Using the VM anode location with the drill bit at station III, the applied current was varied based on previous studies 6 examining the relationship between the necessary current supplied to evoke a response and the distance to the nerve root. Results for the 2 mm and 10 mm positions showed peak responses within ±4% of the results at 5 mm. Similarly, holding the applied current constant at 8 mA resulted in an increasing fraction of the nerve root surpassing a threshold level of 0.1 mA/cm^2^ as the drill was positioned closer to S1.

A vector plot of the current density magnitude and direction is shown for anode locations IS (Fig. **5** top) and VM (Fig. **5** bottom) with the drill bit at station II. For the VM configuration, the current flows primarily down the drill bit, then posteriorly and superiorly through S1 and then anteriorly to the anode. The response with the IS anode indicates that, while the current does flow down the drill bit towards the nerve root, a strong component of the current will be short-circuited from drill bit to the anode before reaching the pelvic structure.

## DISCUSSION

The finite element model demonstrates the effects of changing anode location and drill bit position on the current flow through the S1 nerve root. The dip in |Jmax| found near the level of drill bit insertion is due to current being shunted away from the S1 nerve root, passing instead through the conductive soft tissue surrounding the root in the S1 foramen. Moving either in the inferior or superior direction from the drill bit, the soft tissue current traveling parallel to S1 re-enters the nerve root as the foramen narrows, as opposed to the insulating bone, thus leading to the peak responses approximately 1 cm inferior and superior to the insertion plane. The actual peak |Jmax|, as shown in Fig. (**3**), occurs along the superior pathway due to both anatomical difference in conductivity as well as the fact that the anode locations are at the level of the umbilicus, superior to the drill bit insertion plane.

For a given stimulus, the DM, VM, and CASIS anode locations were the most effective at producing large current density magnitudes in the nerve root. The similar peak current densities produced in the VM and CASIS positions will yield equivalent current thresholds and, consequently, their monitoring effectiveness will be comparable. Use of the DM anode location would lead to an artificially low current threshold, due to the increased peak level of |Jmax|, thus increasing the likelihood of eliciting an EMG response and producing an underestimation of the drill bit to nerve root distance. Therefore, in the clinical situation, the DM anode location should err on the side of safety, as the drill bit would be farther from the nerve root than expected. The attenuated response seen with the IASIS and IS anode locations makes their clinical implementation risky since a much larger current threshold would be needed to evoke a response, potentially leading to an artificially large inferred drill bit to nerve root distance [10]. Functionally, when using the IASIS or IS configurations, the drill bit would be much closer to the nerve root than expected.

**Fig. (4):**
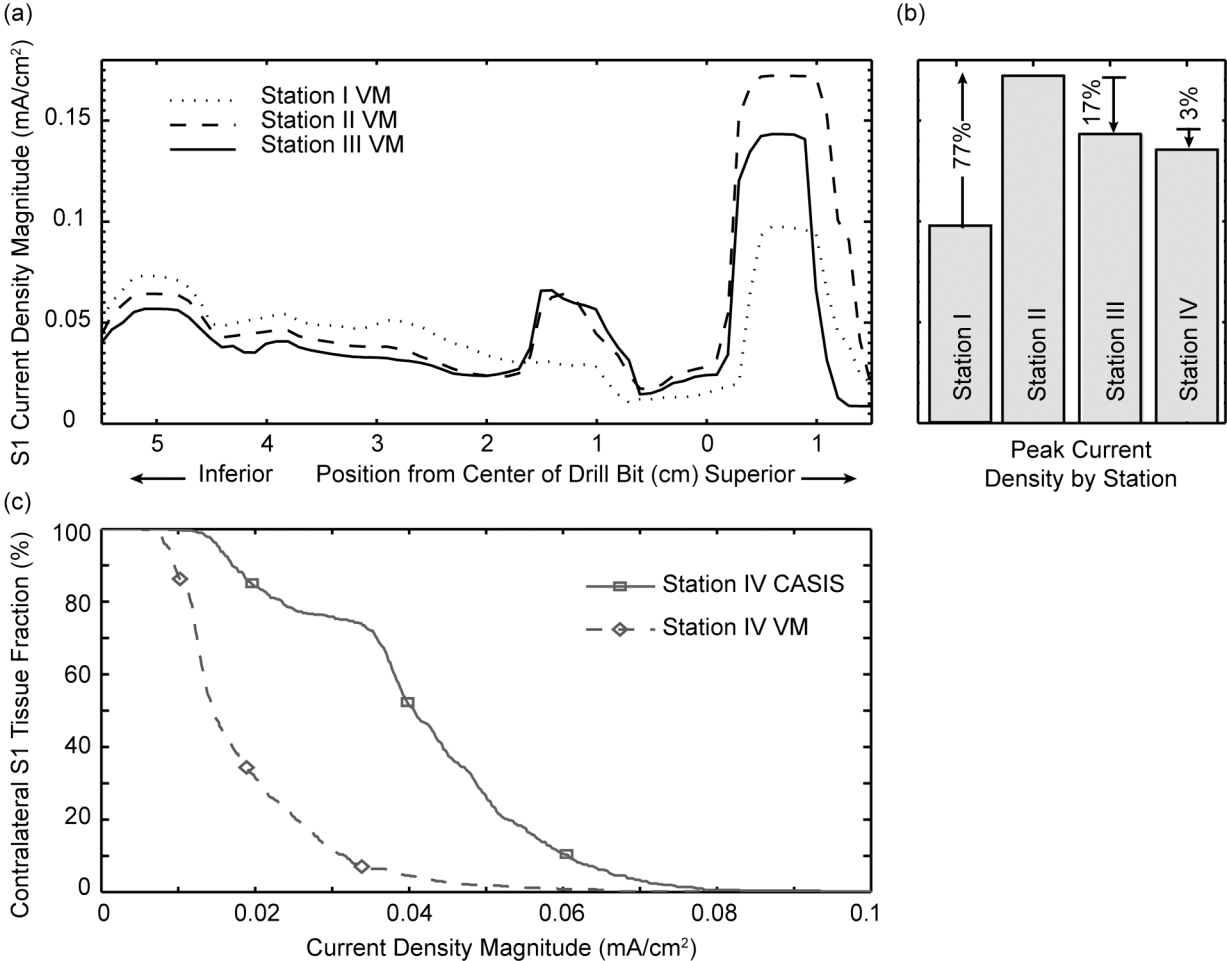
**(a)** Current density plotted versus axial position for three drill bit insertion stations. The drill bit was 5 mm from the nerve root and the anode was located at the VM position. **(b)** Comparison of peak current density |Jmax| at all four insertion sites. **(c)** Backward-cumulative histogram of the contralateral S1 tissue fraction versus current density magnitude for the VM and CASIS anode locations.

**Fig. (5):**
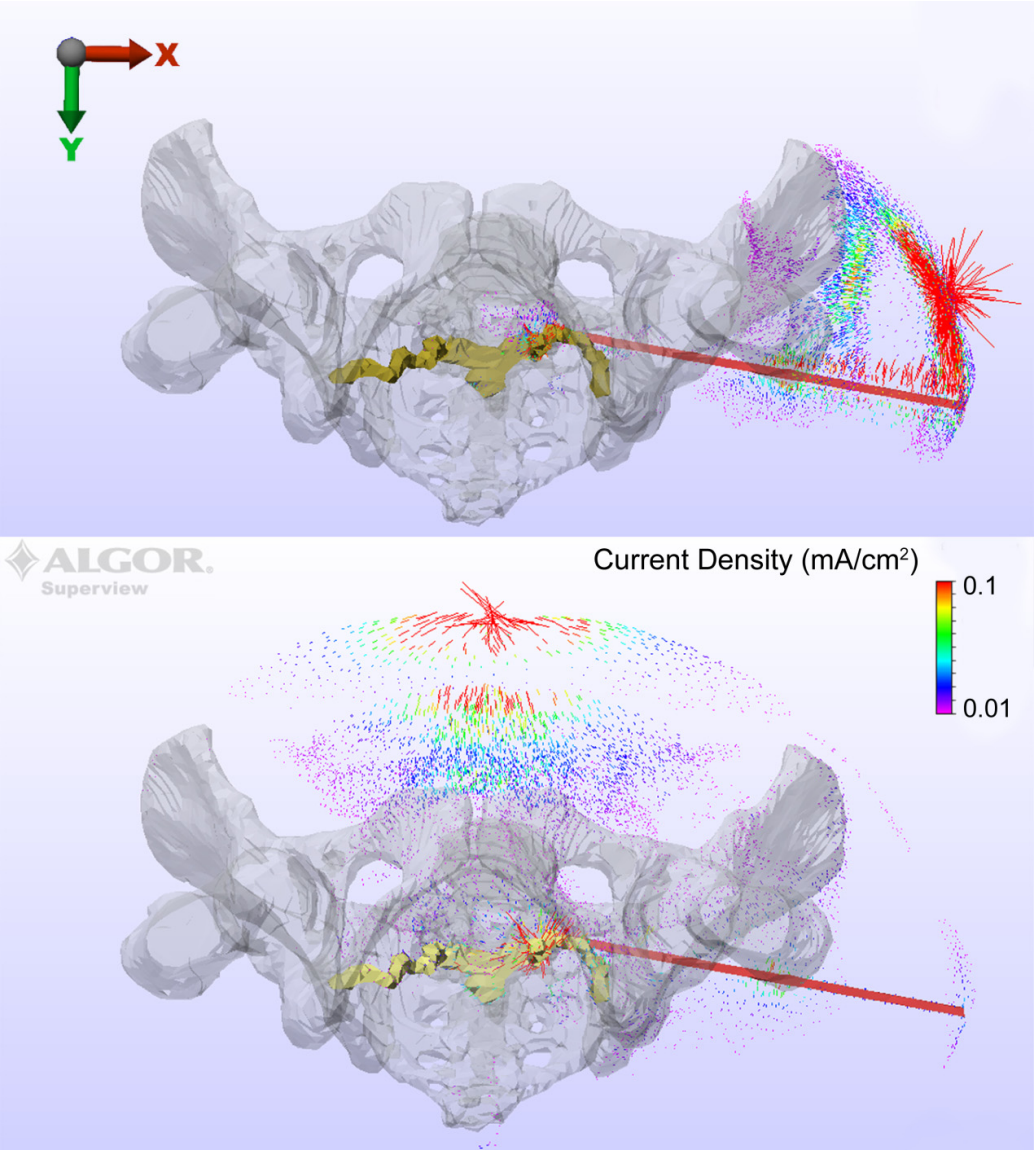
Current density magnitude and direction is shown for anode locations IS (top) and VM (bottom) with the drill bit at station II.

As the drill bit moves into the bone towards its final resting place (Fig. **4**), the results indicate a decrease in peak current density moving from station II to III, with the maximum current in S1 occurring just as the drill bit passes anteromedial to the nerve root. As the tip of the drill bit continues to move past the nerve root, the current will tend to flow down the highly conductive drill bit into the bone rather than enter the nerve root. Consequently the current threshold will rise as the nerve is passed, but the impact is minimal since the risk to the ipsilateral root no longer exists. However, there is an additional hazard to the contralateral root should insertion proceed past the midline. As the drill bit approached the contralateral nerve root (station IV), moving the anode to the CASIS location increased current density significantly. These data support the contention that moving the anode to the CASIS location once the drill bit has crossed the midline is beneficial to monitoring the contralateral nerve [10].

As the perpendicular distance between the drill bit away and nerve is increased, the current required to evoke a response is increased. Table **2** illustrates that the current must be increase as the distance increases in order to maintain similar peak levels; and that maintaining the same current level as the distance increases leads to a decreasing fraction of the nerve root at a specific threshold current density level. In this case, the threshold current density was taken as 0.1 mA/cm^2^, a level consistent with threshold values required for the stimulation of 20 μm fibers by low frequency fields (<1 kHz) [21, 22]. Overall, the strength and duration of the stimulus waveform, fiber size, as well as the electrode size, material, and location all factor into the absolute threshold value [23-26]; however, qualitatively it is clear that the percentage of nerve root reaching a particular level is increased as the distance from the drill bit is decreased.

The anatomical structure and tissue conductivities used within the model lead to a complex three-dimensional distribution of current that clearly impacts the final results. Despite the nerve root being posterior to the drill bit, the results in Fig. (**5** top) show the VM anode performs adequately in providing a pathway for current to flow through the nerve root to the anode. In contrast, Fig. (**5** bottom) indicates that the IS anode location leads to a short circuit behavior when injecting current through the drill bit. The remaining current that does flow to the S1 nerve root is attenuated significantly, thus leading to the predicted overestimation of the distance between root and drill bit [10].

The validity of the relationship between the stimulus-evoked electromyographic current threshold and the distance between the wire and the nerve and its potential applicability for nerve monitoring has been confirmed in both animal and clinical studies [6-8]. In an initial animal study, Moed *et al.* produced findings in a canine model indicating that that stimulus-evoked EMG monitoring during the placement of iliosacral implants was feasible and could be helpful to avoid iatrogenic injury to a lumbosacral nerve root [7]. Subsequently, this conclusion was further substantiated by these investigators in an clinical series of twenty-seven patients [6]. Further animal study showed stimulus-evoked EMG monitoring to be superior to either somatosensory evoked-potential or spontaneous electromyographic monitoring for the purpose of minimizing nerve-root injury during the insertion of iliosacral implants [8].

For many years, stimulus-evoked electromyography has been used successfully during operations on the facial nerve and the recurrent laryngeal nerve [27-31] and during pedicle-screw fixation of the lumbosacral spine in both animals and humans [32-35]. Although intraoperative nerve-monitoring can be expensive (reported to be in excess of $400 per hour at some institutions), the addition of stimulus-evoked electromyography to a system for monitoring somatosensory evoked potentials or continuous electromyography involves no cost, no specific institutional or government approvals, and is easily implemented [6].

## CONCLUSIONS

Based on this study, the CASIS, VM, and DM anode locations provide adequate monitoring of the lumbosacral nerve roots during iliosacral screw insertion; the IASIS and insertion site locations are much less effective in placing the monitored neural structure in the path of current flow. Furthermore, in every screw placement procedure there should be an incremental rise in current threshold just as the drill bit passes anteromedial to the nerve root. This study indicates that while the anode location is clearly an important variable in nerve monitoring, the results obtained at anode locations that place the nerve root outside of the primary electric field can be anticipated. Modification of the present stimulus evoked electromyography monitoring technique to track current thresholds at the DM, the VM, and the CASIS anode locations simultaneously would seem to be the next logical step in reducing the incidence of nerve root injury, since each gives different data about the proximity to the neural structure in question. Further analyses that incorporate optimized anode location or examine nerve monitoring techniques in other regions of the body may be avenues for future research.

## Figures and Tables

**Fig. (1):**
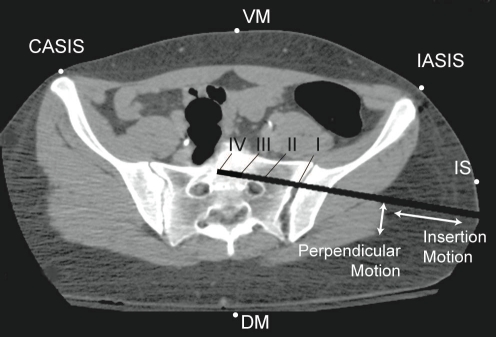
A CT image showing the five simulated anode locations and four drill bit positions of surgical interest along the path described as insertion motion. Translation of the drill bit from the nerve root towards the anterior cortex of the sacrum, identified as perpendicular motion, is also modeled at three distances (2 mm, 5 mm, and 10 mm).

**Fig. (2):**
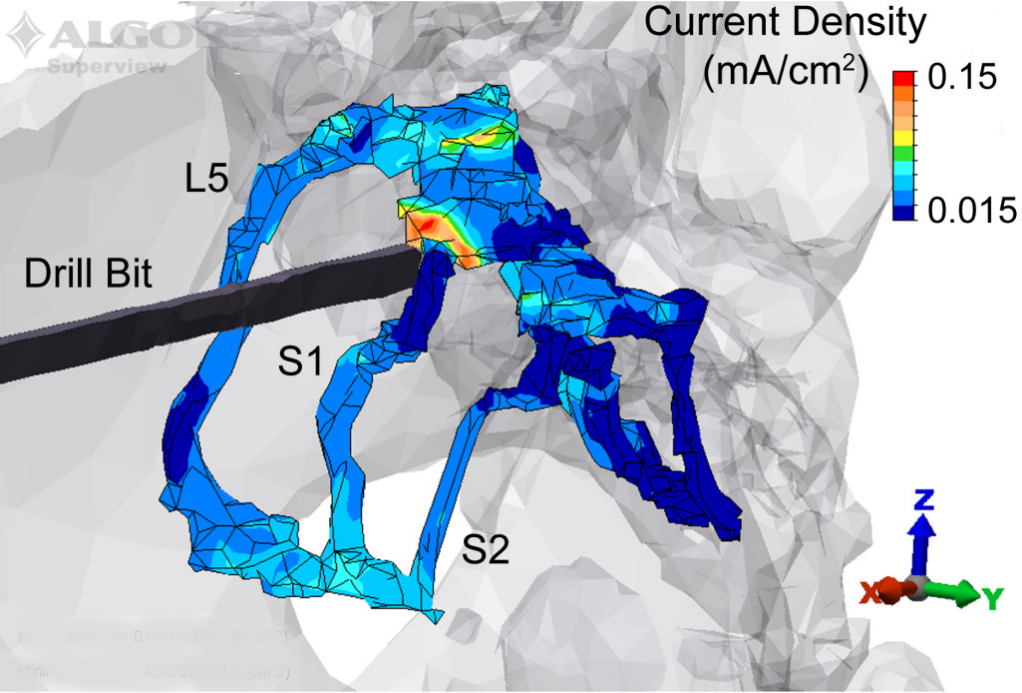
A posterolateral view of the 5 mm model with the drill bit located at station III, and the anode placed on the ventral midline. The current density in the L5, S1 and S2 nerve roots is displayed along with the structural outline of the bone and drill bit. The peak current density occurs in the S1 nerve root immediately posterior and superior to the drill bit.

**Fig. (3):**
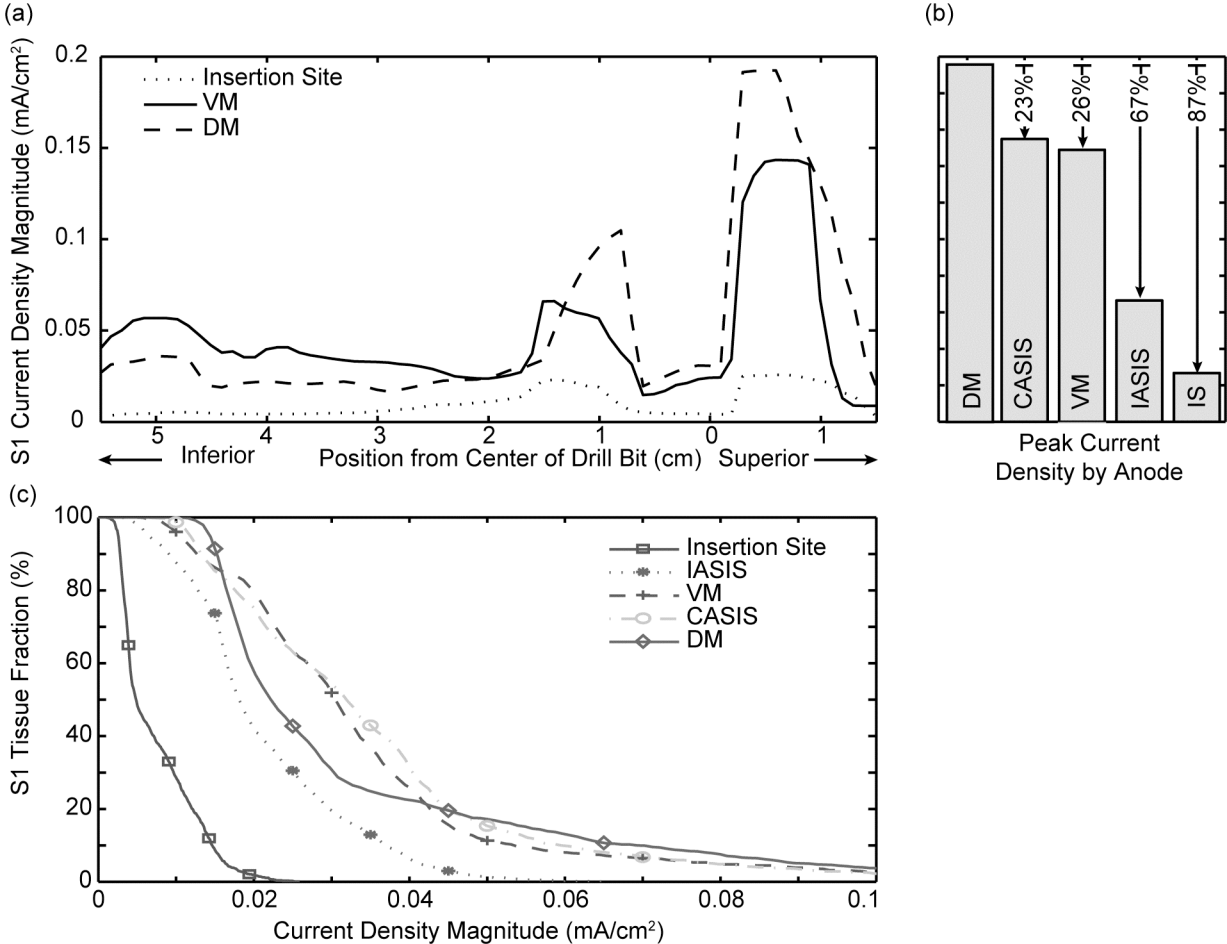
**(a)** Current density plotted versus axial position for three anode locations. The drill bit was 5 mm from the nerve root and located at station III. The DM location exhibited the largest current density. **(b)** Comparison of peak current density |Jmax| for all five anode locations. **(c)** Backward-cumulative histogram depicting the fraction of S1 versus current density magnitude.

**Table 1.** Model Element Count and Conductivity Data by Tissue Type

Tissue	Conductivity (S/m)	Number of Elements
Fat	0.0400^a^	86751
Intestine	0.1000^a^	26021
Isotonic Soft Tissue	1.20000	13245
Muscle	0.1050^a^	105309
Bone	0.0057^b^	74759
Vasculature (Whole blood)	0.6250^c^	5672
Nervous Tissue	0.1736^d^	50829
Vertebral Disc	0.7355^e^	659
Cerebrospinal Fluid	1.4580^d^	14284
Bladder (Urine)	3.0000^f^	3273
Drill Bit (Ti6Al4V)	571600	2822
Boundary and Current Source Elements		70
Total		383694

a See Schwan and Kay [15]

b Averaged from Saha and Williams [16]

c See Geddes and Sadler [17]

d See Geddes and Baker [18]

e See Gu *et al*. [19]

f See Bichonski and Pawelek [20]

The number of elements shown is for the model with drill bit to nerve root distance of 5 mm, station III of its insertion path, and the VM anode location.

**Table 2.** Response to Drill Bit Translation Perpendicular to the Direction of Insertion

Perpendicular Distance from Drill Bit to S1	Applied Current (mA): Peak Current Density (mA/cm^2^)	Fraction of S1 Above 0.1 mA/cm^2^ for Fixed 8 mA Applied Current
2 mm	4.71 : 0.096	8.1%
5 mm	7.28 : 0.098	3.8%
10 mm	10.78 : 0.102	0.2%
